# Application of calcium-to-phosphorus (Ca/P) ratio in the diagnosis of pseudohypoparathyroidism: another piece in the puzzle of diagnosis of Ca-P metabolism disorders

**DOI:** 10.3389/fendo.2023.1268704

**Published:** 2023-10-03

**Authors:** Sara De Vincentis, Giulia Del Sindaco, Angela Pagnano, Giulia Brigante, Antonio Moretti, Lucia Zirilli, Vincenzo Rochira, Manuela Simoni, Giovanna Mantovani, Bruno Madeo

**Affiliations:** ^1^ Endocrinology, Department of Biomedical, Metabolic and Neural Sciences, University of Modena and Reggio Emilia, Modena, Italy; ^2^ Unit of Endocrinology, Department of Medical Specialties, Azienda Ospedaliero-Universitaria di Modena Policlinico di Modena, Ospedale Civile di Baggiovara, Modena, Italy; ^3^ Clinical and Experimental Medicine PhD Program, University of Modena and Reggio Emilia, Modena, Italy; ^4^ Department of Clinical Sciences and Community Health, University of Milan, Milan, Italy; ^5^ Endocrinology Unit, Fondazione IRCCS Ca’ Granda Ospedale Maggiore Policlinico, Milan, Italy

**Keywords:** hypocalcemia, hyperphosphatemia, hypoparathyroidism, parathyroid dysfunctions, mineral disorders

## Abstract

**Objective:**

The serum calcium (Ca)–to–phosphorus (P) ratio has been proposed to identify patients with primary hyperparathyroidism and chronic hypoparathyroidism (HPT), but it has never been tested in pseudohypoparathyroidism (PHP). The aim of this study was to test the performance of Ca/P ratio in PHP diagnosis compared with that in healthy subjects and patients with HPT for differential diagnosis.

**Design:**

A retrospective, cross-sectional, and observational study was carried out.

**Methods:**

Serum Ca, P, creatinine, parathyroid hormone (PTH), and albumin were collected. Ca and P were expressed in mmol/L. Ca/P diagnostic performance was evaluated by receiver operating characteristic curve, sensitivity, specificity, and accuracy.

**Results:**

A total of 60 patients with PHP, 60 patients with HPT, and 120 controls were enrolled. The Ca/P ratio was lower in patients with PHP and HPT than that in controls (p < 0.0001). The cutoff of 1.78 (2.32 if Ca and P measured in mg/dL) for Ca/P ratio could identify patients with PHP and HPT among the entire cohort (sensitivity and specificity of 76%). No valid cutoff of Ca/P was found to distinguish patients with PHP from patients with HPT; in this case, PTH above 53.0 ng/dL identified patients with PHP (sensitivity and specificity of 100%). The index (Ca/P × PTH) above 116 ng/L recognized patients with PHP from controls (sensitivity of 84.7% and specificity of 87.4%), whereas (Ca/P × PTH) below 34 ng/L recognized patients with HPT from controls (sensitivity of 88.9% and specificity of 90.8%).

**Conclusions:**

The Ca/P ratio below 1.78 (2.32 CU) is highly accurate to identify patients with PHP and HPT, although it is not reliable to differentiate these two conditions. The index (Ca/P × PTH) is excellent to specifically recognize PHP or HPT from healthy subjects.

## Introduction

The serum calcium (Ca)–to–phosphorous (P) (Ca/P) ratio has been proposed as a simple tool to detect disorders of Ca and P metabolism such as primary hyperparathyroidism (PHPT) and chronic hypoparathyroidism (HPT) (1–4). In detail, it has been demonstrated that the serum Ca/P ratio is highly accurate in detecting PHPT when above 2.55 (3.3 if serum Ca and P are expressed in mg/dL) and HPT when below 1.78 (2.32 if serum Ca and P are expressed in mg/dL) (3).

The rationale of the usefulness of the Ca/P ratio lies in the inverse relationship between serum Ca and P that leads to high serum Ca coupled with low serum P or *vice versa*, as traditional biochemical profiles of PHPT and HPT, respectively. Similarly, other less frequent parathyroid dysfunctions are theoretically characterized by the same biochemical pattern of PHPT and HPT, for which the use of serum Ca/P could be extended. For example, non-PTH–mediated hypophosphatemia might be detected using the same cutoff of serum Ca/P that is suggestive for PHPT (2).

Pseudohypoparathyroidism (PHP) includes a constellation of disorders that share biochemical characteristics of HPT (i.e., hypocalcemia and hyperphosphatemia), as a result of proximal tubular resistance to parathyroid hormone (PTH) (5, 6). In contrast to HPT, PTH resistance is the hallmark of PHP, defined by elevated serum levels of PTH beyond hypocalcemia and hyperphosphatemia (6, 7). Patients with PHP may be associated with a similar spectrum of physical abnormalities such as short stature, brachydactyly, obesity, cognitive impairment, and heterotopic ossifications, collectively referred to as Albright hereditary osteodystrophy (5). Some patients present with resistance to other hormones (e.g., thyroid-stimulating hormone and/or gonadotropins, growth-hormone-releasing hormone, and calcitonin) (5). Hence, diagnosis of PHP should be based on clinical and biochemical characteristics and, in some cases, on the family history (5, 6). Once a clinical suspicion exists, molecular analyses are critically important for genetic counseling and for diagnosis. According to the common pathophysiology and the newly proposed nomenclature and classification, this disorder is included in the group of inactivating PTH/Parathyroid hormone-related peptide (PTHrP) signaling disorders (iPPSD): in particular, PHP type 1A or iPPSD2 is due to maternal loss-of-function variants at the *GNAS* coding sequence (PHP1A), and PHP type 1B or iPPSD3 is due to methylation defect at the GNAS locus (PHP1B) (5, 6, 8). Subtypes of PHP share a common defect in the cAMP signaling pathway downstream of the PTH/PTHrP receptor. Despite this unifying molecular umbrella, presentation and disease severity can vary considerably between affected individuals, even among patients carrying the same genetic alteration (9). Diagnosis may be difficult in the absence of one or several biochemical features (hypocalcemia or hyperphosphatemia) (9, 10), also considering that PTH resistance is usually absent at birth and develops over time (11). A subset of affected subjects are not diagnosed until adulthood, especially those with PHP1B subtype, and some individuals have no evidence for hypocalcemia or secondary hyperparathyroidism, when evaluated later in life (12). For this reason, the serum Ca/P ratio could potentially help in the diagnostic workup of PHP, especially in asymptomatic cases or in those who are starting to show a progressive increase in PTH levels with still normal Ca and P. However, no study has hitherto investigated the accuracy of Ca/P ratio as diagnostic tool in patients with clinical suspicion of PHP. The aim of this study is to explore the diagnostic power of the serum Ca/P ratio in the diagnosis of PHP in comparison with that in healthy subjects and in patients with HPT for differential diagnosis.

## Subjects and methods

### Study design

A multicentric, retrospective, and cross-sectional study was carried out.

Enrolled patients were subdivided in three subgroups: patients with documented PHP, patients with HPT, and subjects without any known disorder of Ca-P metabolism.

### Subjects

Patients with a documented diagnosis of PHP and HPT from January 2005 to January 2018 were retrospectively recruited from the Endocrinology Units of Fondazione IRCCS Ca’ Granda Ospedale Maggiore Policlinico in Milan and University of Modena and Reggio Emilia, respectively. HPT diagnosis was provided in agreement with the current related guidelines (13). PHP diagnosis was confirmed by genetic test in almost all cases. Moreover, we retrospectively selected 120 patients who attended the Unit of Endocrinology of Modena in the same period of time for other endocrine diseases not related to Ca-P metabolism and presenting serum PTH, Ca, and P within the normal range.

The following parameters were collected from patients’ record charts for the inclusion in each group: age, gender, serum Ca, P, PTH, and creatinine (**Table 1**).

**Table 1:** Age, gender, and biochemical differences between patients with PHP and HPT and controls. Measurements are expressed as median [Interquartile Range (IQR)].

	Normal range	PHP *n* = 60	HPT *n* = 60	Controls *n* = 120	p-value
Age					
(years)	**-**	38.9 (30.2–44.8)		41.0 (28.7–47.3)	0.454
		35.1 (27.5–41.1)*°	42.4 (34.9–46.4)		**0.010**
Sex					
Females (%)	**-**	72 (60.0)		66 (55.0)	0.514
		31 (51.7)	41 (68.3)		
Males (%)	**-**	48 (40.0)		54 (45.0)	
		29 (48.3)	19 (38.7)		
Biochemical parameters					
**Serum Ca** (mmol/L)	2.12–2.63	1.89 (1.56–2.20)*^#^*	2.05 (1.96–2.18)^	2.38 (2.30–2.43)	**<0.0001**
**Serum P** (mmol/L)	0.81–1.65	1.37 (1.20–1.83)*^#^*	1.37 (1.20–1.51)^	1.16 (1.00–1.31)	**<0.0001**
**Ca/P**	–	1.40 (0.84–1.81)*^#^*	1.52 (1.29–1.76)^	2.04 (1.81–2.30)	**<0.0001**
**Serum PTH** (ng/L)	15–88	264 (125–425)*^# §^*	10.1 (5.3–15.0)^	31.6 (23.0–41.7)	**<0.0001**
**Ca/P** × **PTH** (ng/L)	–	285.4 (176.7–477.8) *^# §^*	18.3 (6.8–25.2)^	63.8 (44.5–87.4)	**<0.0001**
**Serum creatinine** (µmol/L)	44.2–106.1	79.6 (61.9–88.4)	73.4 (59.2–84.0)	70.7 (61.9–79.6)	0.208
**Serum albumin** (g/L)**	35–50	43 (38–46)	41 (40–43)^	44 (41–46)	**<0.0001**
**Serum-corrected Ca** (mmol/L)**	–	1.90 (1.55–2.14)*^#^*	2.05 (1.95–2.14)^	2.30 (2.23–2.35)	**<0.0001**
Corrected Ca/P Ratio**	–	1.51 (1.10–1.84)*^#^*	1.49 (1.28–1.70)^	2.00 (1.75–2.23)	**<0.0001**

PHP, pseudohypoparathyroidism; HypoPT, hypoparathyroidism; Ca, serum calcium; P, serum phosphorus; Ca/P, serum calcium–to–phosphorus ratio; **available in 41 patients with PHP (68.3%), 58 patients with HypoP (96.7%), and 98 controls (81.7%). * PHP vs. controls, p = 0.021; ° PHP vs. HypoPT, p = 0.003; ^#^ PHP vs. controls, p < 0.0001; ^ HypoPT vs. controls, p < 0.0001; and ^§^ PHP vs. HypoPT, p < 0.0001. P-values reaching significance (<0.05) are reported in bold.

For patients with PHP and HPT, parameters were preferably recorded without any ongoing specific treatment (**Table 1**). Because oral integration with Ca and/or vitamin D analogs is routinely administered in case of hypocalcemia even before diagnosis, the enrolment of patients with PHP and HPT without ongoing therapy is difficult. However, PHP and chronic HPT (at least 6 months after neck surgery in case of post-surgical form) on therapy are recommended to maintain serum calcium levels to the lower normal range as to avoid hypercalciuria in concomitance with serum P in the highest limit of the normal range. Thus, biochemical parameters of patients with PHP and HPT under treatment, with no available data before, were collected anyway; in these cases, the abovementioned parameters were obtained at the patient’s lowest calcium level.

Exclusion criteria were as follows: age younger than 18 or older than 90 years; severe renal disease (i.e., estimated glomerular filtration rate <30 mL/min); sever liver insufficiency; active metabolic bone disease e.g., bone Paget’s disease, osteomalacia, and rickets); malnutrition; severe obesity [Body Mass Index (BMI) >40 kg/m^2^]; a history of gastrointestinal malabsorption; sarcoidosis; hypercortisolism, diabetes insipidus, and hyperthyroidism; familial hypocalciuric hypercalcemia; and ongoing treatment with the following drugs: steroids, thiazides, phosphate binders, lithium, cinacalcet, bisphosphonates, teriparatide, and denosumab. Ongoing treatment with native vitamin D was not an exclusion criterion.

### Laboratory analyses

Serum Ca and P were detected by commercially available kits using photometric methods, and they were expressed in mmol/L. Serum intact PTH was determined by second-generation chemiluminescent immunoassays.

Serum albumin was collected when available, although this parameter was not mandatory as inclusion criteria. Plasma albumin–adjusted corrected Ca (corrected Ca) was calculated to account for false low–serum calcium values due to hypoalbuminemia. We applied the following formula to all patients for which serum albumin was available in the record chart: corrected total calcium (mg/dL) = total serum calcium (mg/dL) + 0.8 * [4 − serum albumin (g/L)] (14, 15).

Serum Ca/P ratio was calculated as follows: Ca/P = Ca(mmol/L)/P(mmol/L). To convert International System of Units (SI; mmol/L) to Conventional Units (CU; mg/dL), Ca was multiplied by 4 [i.e., (mg/dL) = 4 (mmol/L)] and P by 3.1 [i.e., (mg/dL) = 3.1 (mmol/L)].

### Ethics

This study was conducted in accordance with the ethical standards of the Helsinki Declaration (1975, revised in 2013), and the study protocol was approved by the North Emilia Vast Area (AVEN) Ethics Committee (protocol number AOU 0036075/21). Considering the retrospective study design, an informed consent was waived by the AVEN Ethics Committee.

### Statistical analysis

According to data distribution analyzed by the Kolmogorov–Smirnov test, comparisons of continuous variables were performed using the nonparametric Mann–Whitney test and Kruskal–Wallis test followed by the Dunn’s multiple comparisons *post-hoc* test when a significant difference was found to establish differences between individual groups. All the data are shown as median and minimum-maximum.

Categorical variables were compared with that in the Chi-square test.

The diagnostic accuracy of Ca/P was investigated using receiver operating characteristic (ROC) curve to define cutoff points that better identify affected patients according to their biochemical profile. ROC cutoffs were calculated by the Youden’s index through the identification of the best pair of sensitivity and specificity.

For each biochemical parameter, sensitivity, specificity, positive predictive value (PPV), negative predictive value (NPV), and accuracy were calculated after defining true and false positives/negatives according to the upper and lower limits of the normal ranges of each laboratory of the participant centers.

Statistical analyses were performed using the Statistical Package for the Social Sciences’ (SPSS) software for Windows (version 27.0; SPSS Inc, Chicago, IL). For all comparisons, p < 0.05 was considered statistically significant.

## Results

A total of 240 patients were enrolled, subdivided into 60 patients with PHP, 60 patients with HPT, and 120 controls according to inclusion and exclusion criteria. In our PHP series, 57 of the 60 patients had a confirmed genetic diagnosis, with 14 being patients with PHP1A and 43 being patients with PHP1B. Considering HPT subgroup, 55 of the 60 patients (91.7%) had a chronic post-operative HPT, whereas only five patients (8.3%) had an idiopathic form of HPT. Age, sex, and biochemical parameters of each group are summarized in **Table 1**.

Serum Ca was significantly lower in both patients with PHP and HPT compared with that in controls (p < 0.0001) (**Table 1**). Conversely, serum P was significantly higher in both patients with PHP and HPT compared with that in controls (p < 0.0001) (**Table 1**). No difference in serum Ca or P was found between patients with PHP and HPT. As expected, serum PTH was higher in PHP group than other two groups (p < 0.0001), whereas it was significantly lower in patients with HPT compared with that in controls (p < 0.0001) (**Table 1**). The Ca/P ratio was significantly higher in controls compared with that in both patients with PHP (p < 0.0001) and HPT (p < 0.0001), but it did not significantly differ among PHP and HPT groups (**Table 1**).

Focusing on PHP subgroup, patients with PHP1A had higher serum Ca (p < 0.001) and lower serum PTH (p = 0.004) compared with patients with PHP1B; no significant difference in serum P was found (**Table 2**). Consequently, even the Ca/P ratio was significantly higher in patients with PHP1A than that in patients with PHP1B (p = 0.003) (**Table 2**). These findings were confirmed restricting the analysis to those patients without ongoing Ca and/or vitamin D supplementation (six of the 14 patients with PHP1A and 27 of the 43 patients with PHP1B).

**Table 2:** Comparison of biochemical parameters between patients with PHP1A and PHP1B. Measurements are expressed as median (IQR).

	Normal range	PHP1A *n* = 14	PHP1B *n* = 43	p-value
**Age** (years)		38.6 (33.8–43.2)	34.0 (27.3–41.1)	0.182
Sex				
Females (%)	**-**	6 (42.9)	22 (51.2)	0.589
Males (%)	**-**	8 (57.1)	21 (48.8)	
Biochemical parameters				
**Serum Ca** (mmol/L)	2.12–2.63	2.20 (1.97–2.30)	1.75 (1.50–1.98)	**<0.0001**
**Serum P** (mmol/L)	0.81–1.65	1.26 (1.12–1.43)	1.51 (1.20–1.91)	0.069
**Ca/P**	–	1.81 (1.43–2.05)	1.24 (0.83–1.66)	**0.003**
**Serum PTH** (ng/L)	15–88	111 (62–291)	308 (205–454)	**0.004**
**Ca/P** × **PTH** (ng/L)	–	173.9 (111.6–570.7)	360.0 (242.5–477.8)	0.171
**Serum creatinine** (µmol/L)	44.2–106.1	70.7 (61.9–83.1)	84.0 (59.2–94.6)	0.559

PHP1A,pseudohypoparathyroidism type 1A; PHP1B, pseudohypoparathyroidism type 1B; Ca, serum calcium; P, serum phosphorus; Ca/P, serum calcium–to–phosphorus ratio (Ca and P measured in mmol/L); PTH, serum parathyroid hormone. P-values reaching significance (<0.05) are reported in bold.

Serum albumin was available from 41 patients with PHP (68.3%), 58 patients with HPT (96.7%), and 98 controls (81.7%); it was used to calculate albumin-adjusted Ca (**Table 1**). Similarly to total serum Ca, even albumin-corrected Ca and its ratio to P were different among groups (p < 0.0001). Both corrected Ca and corrected Ca/P were lower in patients with PHP and HPT compared with that in controls (p < 0.0001), whereas no difference was found between the PHP and HPT groups (**Table 1**).

**Figure 1:**
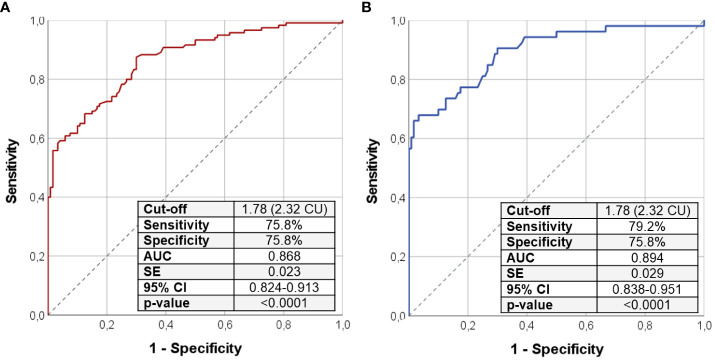
ROC curve analysis using Ca/P ratio to distinguish the entire cohort of patients with PHP/HPT from controls **(A)**; the same analysis was repeated selecting only patients with PHP and HPT without ongoing calcium and vitamin D analog supplementation **(B)**. Sensitivity, specificity, AUC, standard error, and 95% confidence interval values were reported for the most performant Ca/P cutoff that was 2.32 in both cases. Ca/P, serum calcium/phosphorus ratio (Ca and P measured in mmol/L); CU, Conventional Units (Ca and P are measured in mg/dL); AUC, area under the curve; SE, standard error; CI, confidence interval; PHP, pseudohypoparathyroidism; HPT, hypoparathyroidism.

**Figure 2:**
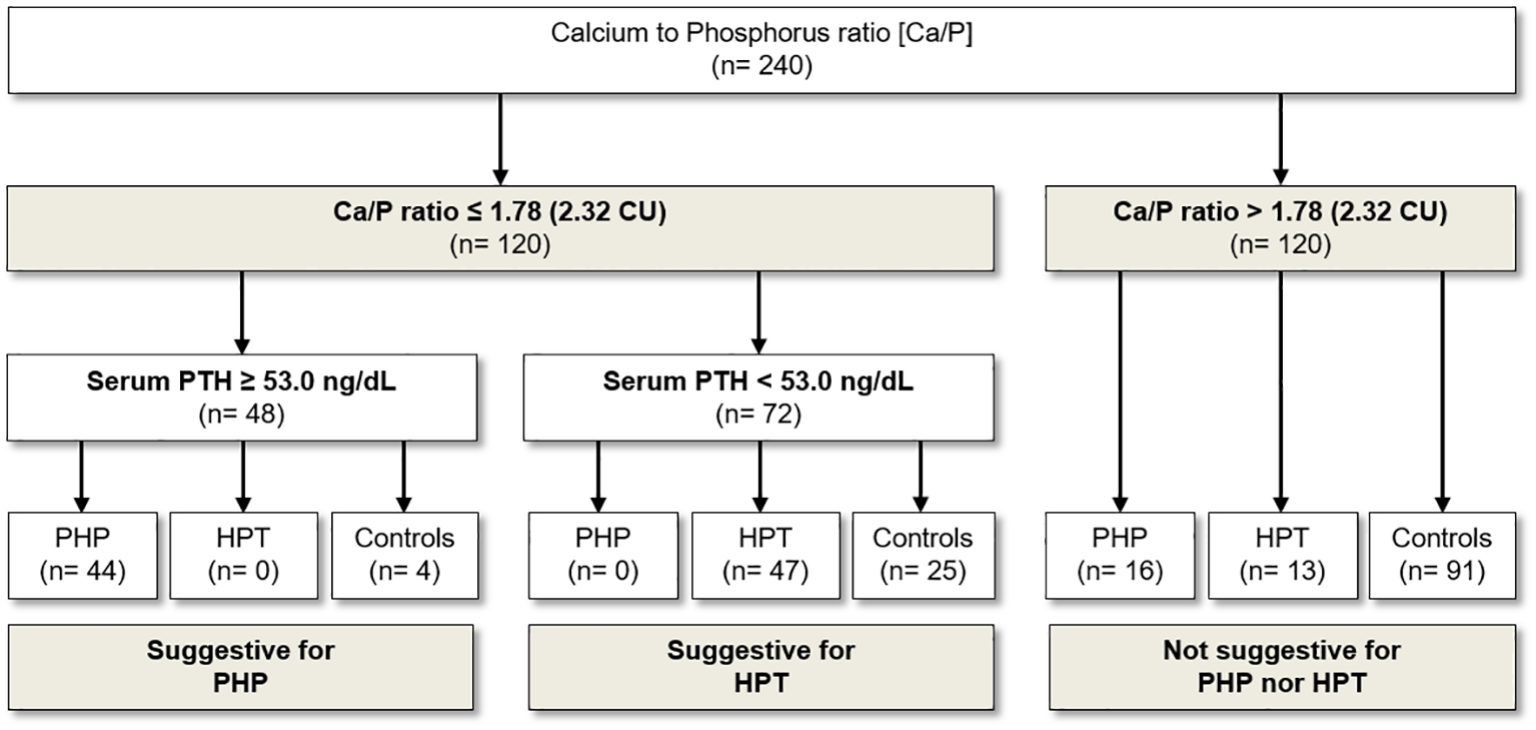
Usefulness of Ca/P in the diagnostic flow chart for the screening and diagnosis of PHP and HPT. Ca, serum calcium; Ca/P, serum calcium/phosphorus ratio (Ca and P measured in mmol/L); PHP, pseudohypoparathyroidism; HPT, hypoparathyroidism; CU, Conventional Units (Ca and P are measured in mg/dL).

The ROC curve analysis identified the cutoff of 1.78 (2.32 if serum Ca and P are expressed in mg/dL) to distinguish patients with PHP and HPT from the healthy subjects with the best pair of sensitivity (75.8%) and specificity (75.8%) (**Table 3**; **Figure 1A**). In particular, a Ca/P ratio below 1.78 allowed the correct identification of 44 of the 60 patients with PHP and of 47 of the 60 patients with HPT, whereas only 29 of the 120 controls were misclassified as affected by an imbalanced Ca-P metabolism (**Figure 2**). When serum albumin was available, the analysis of serum-corrected Ca/P ratio provided similar results (**Table 3**). Notably, both Ca/P and corrected Ca/P ratios showed sensitivity, specificity, PPV, NPV, and accuracy better than serum Ca, corrected Ca, and P alone (**Table 3**). Finally, combining the use of the serum Ca/P ratio with serum Ca (which was considered positive when at least one of the two biochemical parameters was below the respective cutoffs), the sensitivity and the accuracy raised (80.8% and 78.3%, respectively), maintaining excellent specificity and predictive values (**Table 3**).

**Table 3:** Diagnostic value of biochemical parameters for the identification of disorders of Ca-P metabolism (PHP and HPT) in the entire cohort.

	Cutoff	Sensitivity	Specificity	PPV	NPV	Accuracy
**Ca/P**	1.78 (2.32 CU)	75.8	75.8	75.8	75.8	75.8
**Corrected Ca/P ratio**	1.71 (2.22 CU)	78.9	79.2	73.7	83.5	79.0
**Serum Ca** (mmol/L)	2.13 (8.5 CU)	67.5	100	100	75.5	83.8
**Serum-corrected Ca** (mmol/L)	2.13 (8.5 CU)	67.5	100	100	79.7	85.7
**Serum P** (mmol/L)	1.65 (5.1 CU)	23.3	100	100	56.6	61.7
Combined Ca/P and/or serum Ca*	1.78/2.13	80.8	75.8	77.0	79.8	78.3
Combined Ca/P and/or serum P*	1.78/1.65	75.8	75.8	75.8	75.8	75.8

PHP, pseudohypoparathyroidism; HPT, hypoparathyroidism; Ca, serum calcium; P, serum phosphorus; Ca/P, serum calcium–to–phosphorus ratio (Ca and P measured in mmol/L); CU, Conventional Units (Ca and P are measured in mg/dL). Sensitivity: the number of true positives divided by the number of true positives plus the number of false negatives. Specificity: the number of true negatives divided by the number of true negatives plus the number of false positives. PPV, positive predictive value: the number of true positives divided by the number of true positives plus the number of false positives. NPV, negative predictive value: the number of true negatives divided by the number of true negatives plus the number of false negatives. Accuracy: the number of true positives plus the number of true negatives divided by the number of true positives plus the number of true negatives plus the number of false positives plus the number of false negatives. *Combined Ca/P and/or serum Ca was considered positive when at least one of the two biochemical parameters was above the cutoff.

Values are expressed as percentage.

Therefore, we limited the analysis by selecting all patients with Ca-P metabolism disorder (PHP and HPT) with Ca/P below 1.78. As expected, in this setting, the use of serum PTH alone had the best diagnostic value in distinguishing patients with PHP from patients with HPT. In detail, the threshold of 53.0 ng/L for PTH was identified as the most performant (sensitivity of 100% and specificity of 100%) for the detection of patients with PHP and PTH (**Figure 2**). Specifically, all patients with PHP showed serum PTH higher than 53 ng/L, whereas patients with HPT were characterized by serum PTH lower than such threshold (**Figure 2**). With regard to Ca/P ratio, however, no further valid cutoff was found to differentiate patients with PHP from patients with HPT.

**Figure 3:**
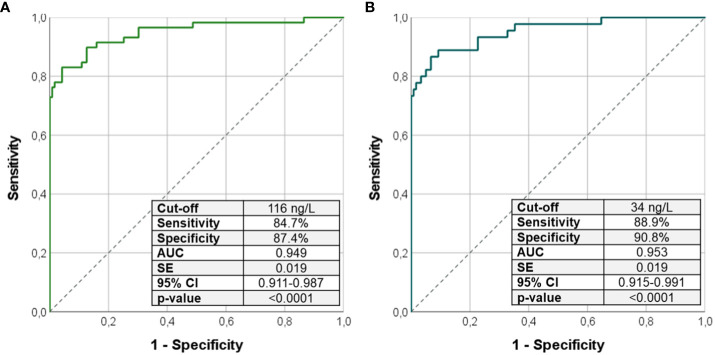
ROC curve analysis using the product (Ca/P × PTH) to identify patients with PHP from controls **(A)** and patients with HPT from controls **(B)**. Sensitivity, specificity, AUC, standard error, and 95% confidence interval values were reported for the most performant cutoffs. Ca/P, serum calcium/phosphorus ratio (Ca and P measured in mg/dL); AUC, area under the curve; SE, standard error; CI, confidence interval; PHP, pseudohypoparathyroidism; HPT, hypoparathyroidism.

Because the use of Ca/P alone was not able to differentiate patients with PHP from patients with HPT, we tested the Ca/P in combination with PTH through the calculation of Ca/P × PTH. (Ca/P × PTH) was higher in PHP (p < 0.0001) and lower in HPT (p < 0.0001) compared with that in controls (**Table 1**). The ROC curve analysis revealed that a Ca/P × PTH was an excellent index to precisely recognize PHP or HPT from healthy subjects (**Figures 3A, B**). In details, (Ca/P × PTH) above 116 ng/L identified patients with PHP among controls (sensitivity of 84.7% and specificity of 87.4%) (**Figure 3A**); on the contrary, (Ca/P × PTH) below 34 ng/L identified patients with PHP among controls (sensitivity of 88.9% and specificity of 90.8%) (**Figure 3B**).

Among PHP and HPT subgroups, 34 of the 60 patients with PHP (56.7%) and 19 of the 60 patients with HPT (31.7%) were free from any treatment with Ca and/or vitamin D analogs. On the other hand, 17 of the 60 patients with PHP and 22 of the patients with HPT received Ca supplementation (mean dosage for PHP, 1475 ± 509 mg; and mean dosage for HPT, 1592 ± 1256 mg); finally, 23 patients with PHP and 39 patients with HPT received calcitriol supplementation (mean dosage for PHP, 0.76 ± 0.36 mg; and mean dosage for HPT, 0.68 ± 0.40 µg).

Assuming that ongoing Ca and vitamin D analog integration may interfere with results, we repeated the same previous analysis including only the 53 patients with PHP/HPT without concomitant confounding therapy. As shown in **Figure 1B**, the cutoff of 1.78 was found to be highly accurate in identifying patients with PHP and HPT among health subjects (sensitivity of 79.2% and specificity of 75.8%) even after the exclusion of patients with ongoing treatment, corroborating the previous findings described for the entire PHP/HPT cohort.

Finally, patients with PHP were younger than both patients with HPT (p = 0.003) and controls (p = 0.021), whereas age did not differ between HPT and controls (**Table 1**). However, taking together patients with PHP and HPT, no difference was found for age compared with controls (**Table 1**). No significant difference was found neither for renal function nor for sex with a prevalence of female subjects in all three groups (**Table 1**).

## Discussion

This study confirms that serum Ca/P ratio is highly accurate in identifying disorders of Ca and P metabolism, showing, for the first time, that it can be applied to detect patients with PHP and corroborating its usefulness for patients with HPT (3). In particular, a Ca/P value below 1.78 (with Ca and P measured in mmol/L) or 2.32 (with Ca and P measured in mg/dL) results to be an accurate, highly sensitive, and specific tool in case of clinical suspicion of PHP/HPT.

These findings are consistent with our previous results (3) because the accuracy of Ca/P below 1.78 to rule in/out a condition of HPT remained unaltered after adding patients with PHP, a setting in which this index has never been tested before. In this study, the validity of the 1.78 Ca/P cutoff was further straightened as it was demonstrated twice by first performing the ROC curve analysis in the entire cohort of patients with PHP and HPT and then restricting the analysis to only those patients free of any calcium and vitamin D supplementation (therefore, theoretically without any confounding treatment).

In addition to HPT group, the inclusion of patients with PHP allowed us to test the usefulness of Ca/P in the differential diagnosis of other Ca-P metabolism disorders with similar biochemical profiles. Unfortunately, we did not find any valid cutoff of Ca/P ratio useful to distinguish patients with PHP from those with HPT. Hence, we can conclude that, whereas the Ca/P below 1.78 is extremely accurate to identify a condition of imbalanced Ca-P metabolism, there is not such a valid cutoff to distinguish patients with PHP from patients with HPT so far. At this step, as expected, the use of PTH was found to be the most performant to differentiate PHP and HPT cases with a threshold of 53.0 ng/L. Interestingly, the combined use of Ca/P × PTH product is able to detect PHP when above 116 ng/dL and HPT when below 34 ng/dL from healthy subjects. Thus, in case of clinical suspicion (e.g., mild hypocalcemia or hyposphatemia), the index (Ca/P × PTH) can be used as valid index to identify a condition of PHP or HPT. This index has already been proposed for the differentiation of primary and secondary hyperparathyroidism (16), but it has never been tested in PHP and HPT setting.

The diagnosis of PHP and HPT can pose challenges because the clinical presentation of both conditions considerably varies with some patients presenting with normal serum Ca at the lowest limit of the normal range or other being totally asymptomatic (5, 17, 18). Precisely for these patients, the serum Ca/P ratio gains value because of its ability to better identify patients affected by disorders of parathyroid function who present a non-classical phenotype such as asymptomatic mild hypocalcemia who currently remain unrecognized (11, 12, 17). The use of serum Ca and P together may facilitate the early identification of PHP and HPT asymptomatic patients, thereby prompting treatment with calcium/vitamin D analogs and preventing the development of severe hypocalcemia and associated *sequelae* (7, 19, 20). Furthermore, in our PHP subgroup, patients with PHP1B were more likely to present a worse biochemical profile at diagnosis compared with patients with PHP1A, characterized by lower serum Ca, higher serum PTH, and, consequently, lower Ca/P ratio, with no difference for serum P. In the literature, there is evidence that, in patients with PHP, resistance to PTH begins in early childhood, whereas the resultant biochemical abnormalities and clinical manifestations typically become apparent later (7, 11, 21). In addition, data suggest that variable degrees of PTH-resistant hypocalcaemia can occur despite identical epigenetic changes (7, 12, 22). However, little is known about the degree of changes in serum Ca and P levels in relation to the different subtypes of PHP. Unpublished data suggest that patients with PHP1B may present with a more severe hypocalcemia at diagnosis compared with patients with PHP1A, in line with our findings.

There is actually claim about the use of serum P as first-step examination in the diagnostic workup of bone mineral diseases and the missed use of diagnostic tests (e.g., serum PTH measurement and definition of hypocalcemia), resulting in frequent underdiagnoses (20). The measurement of serum P, beyond serum Ca, is generally suggested by clinical guidelines and consensus statements on the diagnosis of PHP and HPT, even if it is not considered mandatory (6, 7, 13, 23). Indeed, serum P alone has only a marginal role in the diagnostic workup of these conditions. On the contrary, serum Ca is recognized to have a pivotal role, but even serum Ca alone could be not sufficient. Thus, it is reasonable to suppose that the combined use of serum Ca and P and of their ratio further increases the diagnostic accuracy. Given the findings of this study and the only previous one testing Ca/P in patients with HPT (3), we strongly suggest measuring both serum Ca and P to calculate the Ca/P ratio as the first step in the screening of Ca-P metabolism disorders characterized by hypocalcaemia and hyperphosphatemia, whereas serum PTH is not strictly needed as first-line biochemical investigation.

Accordingly, in contrast to the past literature that was mainly focused on serum Ca (24, 25), the serum P is only recently gaining importance because of a raising body of scientific research that explores the usefulness of the mere phosphate in the management of parathyroid dysfunctions. Castellano et al. observed a significant relationship between P levels and biochemical and clinical features of PHPT severity (26). Interestingly, it has been suggested that, also, in patients with asymptomatic PHPT, even moderate hypophosphatemia is predictive of surgical indication, regardless of age and hypercalcemia severity (26). On the other hand, it should be highlighted that, despite of the potential relationship between serum P and the severity of the disease, the serum P alone is unreliable even in the diagnosis of PHPT, but it should be taken in combination with serum Ca (2, 27–30). For this reason, we could speculate that a similar association between serum P and severity of the disease in terms of long-term complications could be found also in patients with PHP and HPT, although no data are hitherto available about this point. Similarly, the use of Ca/P ratio, which has been tested only as diagnostic index so far, can acquire other important significances from a clinical standpoint, as a predictive marker of complications or as a simple therapeutic target in the management of parathyroid dysfunctions. To date, only the serum Ca and P product is considered a target of therapy in HPT but evidence is scanty (23, 31). However, this issue needs to be specifically investigated in the future aiming at finding good markers for titrating therapy and extrapolating proper Ca/P ratio target references with this purpose.

Because of its worldwide availability and simplicity, this index may be particularly useful in resource-limited health care settings, where the possibility to access genetic counseling and reliable serum PTH assays (i.e., second- or third-generation assays) remains still challenging (6, 13, 20). In terms of cost-effectiveness, its low-cost quality makes the Ca/P ratio an ideal index to be used in the screening of specific subgroups of patients deserving a fast or periodical monitoring of Ca-P balance to rule out altered parathyroid function (e.g., patients after neck surgery, patients with osteoporosis or renal stones, patients with familial history suggestive for Ca-P metabolism disorders, and clinical settings with large inflow of patients such as general practitioners and emergency rooms). In all these settings, the serum Ca/P can help clinicians in early recognizing an impairment of Ca-P balance moving on proper confirmatory tests.

The large sample of such a rare disorder as PHP is the main strength of this study aimed at testing the Ca/P ratio in this setting. The retrospective design represents the main limit of this study. Furthermore, PHP and PHT subgroups are quite heterogeneous due to the presence of patients with ongoing therapy with Ca and vitamin D analogs; however, the analysis restricted to treatment-naïve patients demonstrated that the results were not affected by the ongoing Ca/vitamin D supplementation. Finally, the lack of information about the dietary Ca and P intake could represent a further minor limit of this study.

In conclusion, this study further validates the serum Ca/P ratio below 2.32 (1.78 SI) as a highly accurate tool to identify patients with HPT, and it demonstrates, for the first time, that the same cutoff can be applied for patients with PHP. However, Ca/P ratio alone is not reliable for the differential diagnosis of PHP and HPT. In this case, the combination of Ca/P and PTH through the calculation of (Ca/P × PTH) is an excellent index to specifically recognize PHP (>150 pg/ml) or HPT (<44 pg/ml) from healthy subjects. Because of its extraordinary simplicity, together with the favorable cost-effectiveness, serum Ca and P should be equally considered as first-line examinations to calculate their ratio that can be applied to easily screen/rule out disorders of Ca-P metabolism, especially in asymptomatic patients. Finally, prospective and multi-center studies on larger sample size will be needed to validate our findings and, to some extent, the application of the Ca/P ratio as predictor of long-term complications and therapeutic target in parathyroid dysfunctions.

## Data availability statement

The raw data supporting the conclusions of this article will be made available by the authors, without undue reservation.

## Ethics statement

The studies involving humans were approved by North Emilia Vast Area (AVEN) Ethics Committee. The studies were conducted in accordance with the local legislation and institutional requirements. The participants provided their written informed consent to participate in this study.

## Author contributions

SD: Conceptualization, Data curation, Investigation, Methodology, Software, Writing – original draft, Writing – review & editing. GD: Data curation, Writing – review & editing. AP: Data curation, Writing – review & editing. GB: Data curation, Investigation, Writing – review & editing. AM: Data curation, Writing – review & editing. LZ: Writing – review & editing. VR: Conceptualization, Formal Analysis, Investigation, Supervision, Writing – review & editing. MS: Supervision, Writing – review & editing. GM: Data curation, Investigation, Methodology, Supervision, Writing – review & editing. BM: Conceptualization, Data curation, Investigation, Supervision, Writing – original draft, Writing – review & editing.
